# A Dynamic Energy Budget (DEB) model to describe *Laternula elliptica* (King, 1832) seasonal feeding and metabolism

**DOI:** 10.1371/journal.pone.0183848

**Published:** 2017-08-29

**Authors:** Antonio Agüera, In-Young Ahn, Charlène Guillaumot, Bruno Danis

**Affiliations:** 1 Laboratoire de Biologie Marine CP160/15. Université Libre de Bruxelles, Brussels, Belgium; 2 Korea Polar Research Institute (KOPRI), Yeonsu-gu, Incheon, Republic of Korea; Institute of Zoology, CHINA

## Abstract

Antarctic marine organisms are adapted to an extreme environment, characterized by a very low but stable temperature and a strong seasonality in food availability arousing from variations in day length. Ocean organisms are particularly vulnerable to global climate change with some regions being impacted by temperature increase and changes in primary production. Climate change also affects the biotic components of marine ecosystems and has an impact on the distribution and seasonal physiology of Antarctic marine organisms. Knowledge on the impact of climate change in key species is highly important because their performance affects ecosystem functioning. To predict the effects of climate change on marine ecosystems, a holistic understanding of the life history and physiology of Antarctic key species is urgently needed. DEB (Dynamic Energy Budget) theory captures the metabolic processes of an organism through its entire life cycle as a function of temperature and food availability. The DEB model is a tool that can be used to model lifetime feeding, growth, reproduction, and their responses to changes in biotic and abiotic conditions. In this study, we estimate the DEB model parameters for the bivalve *Laternula elliptica* using literature-extracted and field data. The DEB model we present here aims at better understanding the biology of *L*. *elliptica* and its levels of adaptation to its habitat with a special focus on food seasonality. The model parameters describe a metabolism specifically adapted to low temperatures, with a low maintenance cost and a high capacity to uptake and mobilise energy, providing this organism with a level of energetic performance matching that of related species from temperate regions. It was also found that *L*. *elliptica* has a large energy reserve that allows enduring long periods of starvation. Additionally, we applied DEB parameters to time-series data on biological traits (organism condition, gonad growth) to describe the effect of a varying environment in food and temperature on the organism condition and energy use. The DEB model developed here for *L*. *elliptica* allowed us to improve benchmark knowledge on the ecophysiology of this key species, providing new insights in the role of food availability and temperature on its life cycle and reproduction strategy.

## Introduction

Antarctica includes some of the most challenging habitats on Earth [1]. They are characterised by low temperatures and a very marked seasonality in day length, leading to large variations in ice cover and phytoplankton biomass [2]. Adaptation to such conditions has resulted in organisms generally displaying a poor capacity to cope with temperature elevations [3], yet capable of surviving low-food availability over long periods [4]. These characteristics raise concern about their capacity to face ongoing global climate change. It is now largely accepted that Southern Ocean ecosystems are particularly vulnerable to global warming as some regions are challenged by rapid temperature rise [5,6]. Recent research shows that global warming induces cascading effects, causing a wide variety of changes in the structure and functioning of Antarctic marine ecosystems. The variation in the duration of seasonal sea ice cover, marine-terminating glacier retreat [7], the increase in seasonal ice scouring on sea bottom or highly fluctuating salinity due to glacial melt water introduction [6,8] have for instance been shown to induce changes in key processes for Antarctic ecosystems such as primary production [9] and causing ecosystem structural shifts [10]. Climate change is influencing both physical and biotic components of marine ecosystems, and will have an impact on the distribution and population dynamics of Antarctic marine organisms. Ultimately, life history, distribution and abundance of species reflect the action of metabolic processes in the context of varying environments [11]. To assess the potential effects of climate change on Antarctic benthic marine ecosystems, an in-depth knowledge of metabolic processes is needed. This knowledge will provide a valuable benchmark to quantify species population dynamics, performance, and functional role within a given ecosystem, as well as a ground-truthing ongoing modeling efforts [12].

Dynamic Energy Budget (DEB) theory provides first-principle models describing the processes of energy and matter-uptake and their use for maintenance, development, growth and reproduction of a broad range of organisms [13,14]. DEB theory allows establishing links between the physiology of a model organism and its environment by capturing all the metabolic processes of the organisms through their life cycle as function of matter-uptake and temperature [14]. Derived from DEB theory, DEB models [14] can describe the underlying physiological processes based on first principles (e.g. mass-energy conservation laws, linkage of processes to volume or surface, homeostasis of compounds) [15] common to all life forms. Therefore the DEB model becomes a tool that can be used to model lifetime feeding, growth, reproduction, and their responses to changes in combination of biotic and abiotic conditions [16,17]. The DEB model is a useful tool to fully integrate all organism processes, offering a complete overview of a species physiology and life cycle [17], and its parameters can be used to increase our knowledge on particular processes and adaptations integrating the mechanistic framework underlying the DEB theory [17,18]. This approach addresses the necessity of incorporating species physiology (and actual limitations) in predictive models, which is promising for the description of complex impacts of environmental variations on life history and biological traits.

In this study, we estimated the DEB parameters for *Laternula elliptica*, a large-sized infaunal suspension-feeding bivalve with a circumpolar distribution [19]. *L*. *elliptica* is particularly common in shallow waters (less than 30m) where it is often found in high densities (up to 170 ind.m^-2^ in Marian Cove, King George Is.) in soft sediments, representing a high biomass (289.9 g ash free dry weight m^-2^) [19–21]. *L*. *elliptica* is a key species in Antarctic coastal benthic ecosystems, strongly influencing efficiencies of bentho-pelagic coupling processes [21]. Due to its role in transferring organic carbon from the water column to the benthic realm, *L*. *elliptica* enriches the surrounding sediments, sustaining associated biota [21,22]. Due to its abundance and key-role, the energetic performance of *L*. *elliptica* populations has an important impact on associated ecosystems. *L*. *elliptica* has been broadly used as an experimental model and abundant research literature is available on its growth, gametogenesis, metabolism, feeding, thermal and acidification tolerances [21,23–29]. These studies focus on specific physiological processes, but do not provide an overarching view of the biology/ecology of *L*. *elliptica*. Altogether, these studies describe *L*. *elliptica* as a “typical” Antarctic organism with low metabolic rate, extended lifespan, long larval development, and relatively extended gametogenesis. A few analyses have lead to the development of population models, in an attempt to describe the effect of *L*. *elliptica* population dynamics on its ecosystem [26,30,31]. However, these models do not rely on mechanistic principles, and as such they are limited to describing the observed variability. There are still several gaps in our knowledge of the life history and population dynamics of *L*. *elliptica* such as the role of food and temperature on growth and reproduction, although this bivalve inhabits areas where food availability is highly variable and heavily influenced by environmental conditions such as ice-cover, ice-scouring or land sediment run-off [24,32]. To the best of our knowledge, no comprehensive study has tried to provide a tool to assess how such a variable environment affects *L*. *elliptica* performance and *in extenso* its role within the ecosystem.

The aim of the present study was to use DEB theory to mechanistically describe the adult life cycle of *L*. *elliptica*. Our work provides a quantitative model that can be used to better understand the physiological condition of *L*. *elliptica* and its specific adaptations to its environment. Moreover, the DEB parameters obtained here were explored to delineate the seasonal variability of animal condition exposed to varying environmental conditions of food and temperature. This work responds to a growing need to quantify and predict the effects of environmental changes on the population dynamics of key species from the Southern Ocean. Our DEB model should help to fill some of knowledge gaps in the life history of *L*. *elliptica*. The generality of DEB models will also allow comparisons with closely related temperate and tropical species to confirm or infirm the existence of possibly unique adaptations of the energy budget in this Antarctic species. Moreover, the links established by DEB theory with environmental resources will help to describe the seasonal food availability and its dependencies.

## Material and methods

### Model description

Dynamic Energy Budget (DEB) theory describes the processes of energy and matter-uptake throughout life [14]. The DEB model divides the mass and energy of an organism into four state variables: reserves (*E*), structural volume (*V*), maturity (*E*_*H*_) and reproduction buffer (*E*_*R*_). Energy enters the organism as food (*X*) and is assimilated at a rate of *ṗ*_*A*_ into reserves. The mobilisation rate (*ṗ*_*C*_) regulates the energy mobilised from the reserves to cover somatic maintenance (*ṗ*_*M*_), structural growth (*ṗ*_*G*_), maturity maintenance (*ṗ*_*J*_), maturation (*ṗ*_*R*_) (immature individuals) and reproduction (*ṗ*_*R*_) (mature individuals). *κ* is the proportion of the mobilised energy diverted to *ṗ*_*M*_ and *ṗ*_*G*_, while the rest is used for *ṗ*_*J*_ and *ṗ*_*R*_. In DEB, assimilation is a function of food availability, following a functional response of Holling type II. Mobilisation however depends on the amount of energy stored into the reserves (see S1 File for detailed description of DEB assumptions, schematic representation and notation).

DEB theory assumes isomorphism (animal shape does not change with growth) [14]. The ratio between physical size, structural volume and surface remains constant as the animal grows. Marine benthic animals with pelagic larval stages undergo metamorphosis, which leads to a change in morphometry (or body shape) and therefore our DEB model incorporates one shape coefficient for the adult (*δ*_*M*_) and a different one for the D-larvae (*δ*_*M*.*larv*_). DEB model intends to describe the entire life cycle with the same set of parameters. It has been reported that *Laternula elliptica* has an encapsulated larval stage [33,34], during which the larva develops by consuming egg reserves within the capsule, without external feeding. After hatching out, the D-shaped larva settles on the bottom, starts feeding, and matures into an adult. In this work, we do not intend to describe the larval development of *L*. *elliptica*, however we consider this characteristic to help the model parametrisation with a realistic birth event of a D-larva with a different shape coefficient than that of the adult clam. Although we do not know if the larval development is accelerated, we considered a model parametrisation including an accelerated stage as this is a general characteristic of bivalves [14,35].

### Estimation of DEB model parameters

DEB model parameters are derived from data determined for natural populations and experimental studies where the effects of controlled variables (e.g. temperature or food level) on growth, metabolic rate, reproductive output of individuals were measured [15,36]. There is a vast literature-based knowledge about several aspects of *L*. *elliptica* population dynamics in several localities around Antarctica [23,24,26,37,38]. However, little work has been carried out experimentally, under controlled conditions. In this study, we extracted literature-based data for parameter estimation (see Table 1 for a parameter list and corresponding definition and units). This data was used in combination with data gathered from Marian Cove between 1998 and 1999 on gonad development and animal condition for a single population. Detailed description of this data gathering and processing can be found in Ahn et al. [24].

**Table 1:** DEB parameters values for *Laternula elliptica*. These parameters are given for a temperature of 273.15 K.

Parameter	Symbol	Value	Units
**Basic DEB parameters**			
Maximum structural length^1^	*L*_*m*_	8.426	cm
Maximum surface area-specific assimilation rate ^1^	*{ṗ*_*Am*_*}*	87.752	J d^-1^ cm^-2^
Volume-specific cost of maintenance ^2^	*[ṗ*_*M*_*]*	6.861	J d^-1^ cm^-3^
Volume-specific cost of structure ^1^	*[E*_*G*_*]*	2371	J cm^-3^
Fraction of energy allocated to somatic maintenance and growth ^1^	*κ*	0.659	-
Maturity at birth ^1^	_*$E_{H}^{b}$*_	3.371	J
Maturity at puberty (onset first gametogenesis) ^1^	*$E_{H}^{p}$*	2116	J
Scaled functional response at Marian Cove^1^	*f*_*MC*_	0.332	-
Scaled functional response at Potter Cove^1^	*f*_*PC*_	0.384	-
Scaled functional response at Rothera^3^	*f*_*R*_	0.8	-
**DEB compound parameters**			
Energy conductance ^1^	*$\dot{\nu }$*	0.023	cm d^-1^
Maturity maintenance rate coefficient ^1^	*$\dot{k_{J}}$*	0.001	d^-^^1^
**Shape coefficients**			
Post-metamorphic ^2^	*δ*_*M*_	0.341	-
Pre-metamorphic ^1^	*δ*_*M*.*lrv*_	7.227	-
**Temperature sensitivity**			
Arrhenius temperature ^2^	*T*_*A*_	4832±1306	K
Arrhenius temperature at lower limit ^2^	*T*_*AL*_	19966±1.5x10^5^	K
Lower temperature limit^2^	*T*_*L*_	271±1.74	K
**Conversion parameters**			
Density of structure ^3^	*d*_*V*_	0.09	g cm^-3^
Weight-energy coupler for reserves ^3^	*ρ*_*E*_	4.35x10^-5^	g J^-1^
Molecular weight of reserves ^3^	*w*_*E*_	23.9	g mol^-1^
Chemical potential of reserves ^3^	*${\bar{\mu }}_{E}$*	550	kJ mol^-1^

^1^ Estimated using the covariation method

^2^ Estimated from data.

^3^ Fixed

Starting values for some DEB parameters were obtained directly from experimental studies and field observations, as follows:

#### Temperature sensitivity: Arrhenius parameters

DEB theory integrates the Arrhenius concept of enzyme activation to account for the sensitivity of metabolic rates to temperature [14] (S1 File). Arrhenius temperature (*T*_*A*_) can be calculated from observed values of rates, such as metabolic or growth rates. The DEB model uses a curve for temperature sensitivity given by 5 parameters. However, it is possible to use a three-parameter function considering only the lower limit of the temperature range (S1 File). There were no conclusive data to determine the upper temperature limit parameters for *L*. *elliptica*. Therefore, this study only attempted the parametrisation of the three-parameter Arrhenius function.

Parameters were obtained by adjusting the Arrhenius function to the scaled values on oxygen consumption measured by Peck et al. [39] by means of a non-linear least squares regression using the package minpack.lm [40] and R v.3.15 [41].

#### Post-metamorphic shape coefficient

A post-metamorphic shape coefficient (*δ*_*M*_) was calculated based on the relationship between the body ash-free dry weight (*W*_*d*_) (after subtraction of the weight of the gonad) and the animal’s shell length (*L*_*w*_) by fitting the equation *W*_*d*_ = (*δ*_*M*_ · *L*_*w*_)^3^ by means of a weighted least squares regression [15]. The post-metamorphic shape coefficient was calculated from observations of individuals collected by Ahn et al. [24] in Marian Cove and the data provided by Ahn and Shim [23].

#### The covariation method for DEB parameter estimation

**Fig 1:**
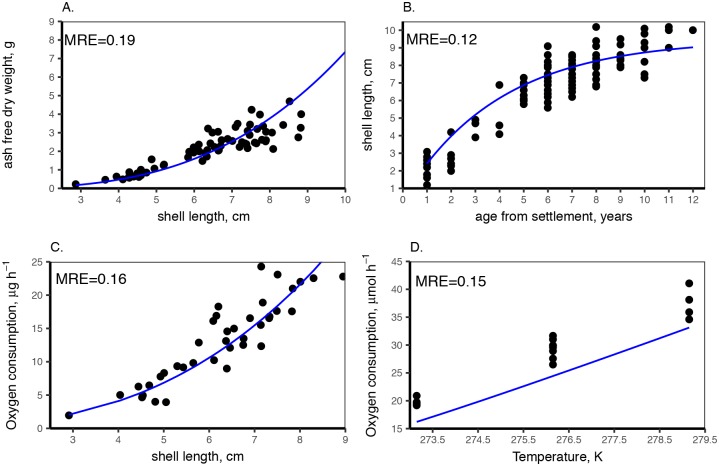
DEB model outputs and uni-variate data used for DEB parameter estimation. **A**. Total ash free dry weight (no gonads) as a function of shell length [23]. **B**. Size at age from shell growth rings at Potter Cove [26]. **C**. Respiration at size at Marian Cove [23] at a temperature of 274.15 K. **D**. Respiration at temperature for a standard individual of 7.5cm shell length [39] excluding assimilation (starved individuals). Dots are data from field observations or laboratory experiments. Blue line represents DEB model output. MRE is the mean absolute relative error.

DEB models are very rich in parameters, however the proportion which can be calculated directly from empirical observations is very limited [15,42]. The covariation method was used for further estimation of the DEB model parameters [42,43] applied with MATLAB^®^ (2015a) using the toolbox package DEBtool (available at http://www.bio.vu.nl/thb/deb/deblab/debtool/). DEB models combine different mechanisms and principles to describe all the processes during the life cycle of an organism [42]. The covariation method uses experimental and field observations of different life stages and approximates the parameters using a Nelder-Mead numerical optimization to minimize the difference between observed and predicted values based on a weighted least-squares criterion [42]. Input data is all connected through the different parameters and the combination of data from different developmental stages and processes at different food availabilities result in a robust prediction of DEB parameters [42]. The parameters previously approximated were used as starting values in the covariation method. The parameters without experimental estimation included pseudo-data, and their starting values were yielded from DEB theory and closely related species [17]. The covariation method is completed with direct observations and data yielded from experiments for which it will approximate the parameters. Two different types of observations can be used in the covariation method [42,43]: zero-variate data represent single data points for a range of different physiological observations; uni-variate data comprise paired data of an independent variable and a dependent variable. The level of fitness of the covariation method is given by the mean absolute relative error (MRE) [44] among all data points and sets used in the parameter estimation. The procedure outputs an MRE value for each zero- and uni-variate variables to help assess the fitness of the predictions to each data set. A list of the zero-variate data used in the estimation of the DEB model parameters can be found in Table 2, zero-variate data corresponding to the population from Marian Cove [23] except for the data on development (age and length at birth) which belong to the population from Rothera station (Marguerite Bay) [34]. Uni-variate data are represented in the results and their origin referenced in Fig 1, and include data from different populations: length-weight from Marian Cove, oxygen comsumption from Rothera and size at age from Potter Cove (King George Island) (S2 File for locations). Using data from different locations allowed to include populations with different condition, feeding at different food levels [17].

**Table 2:** Zero-variate data used for the estimation of the DEB model parameters. MRE: mean absolute relative error.

Variable		Obs.	T (K)	Pred.	Units	MRE	Reference
Age at birth^1^	a_b_	23	274.15	21.6	d	0.06	[34]
Age at puberty^2^	a_p_	730	274.15	557	d	0.23	[49]
D-larva shell length at birth^1^	L_b_	0.02	n/a	0.02	cm	0.03	[34]
Shell length at puberty^2^	L_p_	2.87	n/a	3.19	cm	0.11	[53]
Maximum shell length^3^	L_i_	8.7	n/a	8.19	cm	0.06	[23]
Dry weight at puberty^2^*	dW_p_	0.18	n/a	0.19	g	0.05	[23]
Maximum dry weight^3^*	dW_i_	3.21	n/a	3.19	g	0.01	[23]
Gonadosomatic Index^4^	GSI	0.22	272.7	0.21	-	0.03	[24]

^1^ birth is set at the moment the animal starts or is able to feed. D-larva

^2^ start of first gametogenesis.

^3^ maximum size reached by the species when there is no food limitation. Taken as the upper 95% quantile of population size

^4^ maximum gonad index for an animal of the maximum size, gonad index being defined as gonad weight/total wet weight.

* dry weights correspond to ash free dry weights.

### DEB model parameters application: Describing seasonal metabolism and food availability

Model parameters were applied to describe the seasonal metabolism of *L*. *elliptica* from Marian Cove using the data on animal condition and gonad development during the years 1998 and 1999 [24]. This application aimed at exploring the parameters’ performance on field data that was not used during parameter estimation and additionally to get an insight on the variability of available food for *L*. *elliptica*.

To describe the seasonal changes in energy reserves (*E*) we used observations on shell length and weight after removing the gonad as a proxy to the amount of reserves using DEB parameters (Eq 1) from samples taken during the years 1998 and 1999 (see [24] for details on sampling)

$$E=W-L^{3}$$ (1)

where *W* is weight (g), *L* is structural length (cm, related to physical length through *δ*_*M*_) (Table 1, S1 File). *L*^*3*^ is the structural volume (*V*).

For simplification and easy handling, we used the scaled version of the DEB state variables and we used the scaled energy reserve (Eq 2).

$$e=\frac{W-L^{3}}{E_{m}\cdot L^{3}}$$ (2)

where *e* is the scaled energy reserve (unitless), *E*_*m*_ is the maximum energy density (J cm^-3^) and *L* is again structural length (cm). The denominator is the maximum energy reserve for an organism structural length *L* without food limitation.

Scaled energy reserves were approximated by applying a smoother to the observed values of *e* at each sampling event using the function *gam* from the package mgcv v.1.8–15 [45] and R v.3.15 [41]. The smoother was optimised for a limited number of knots to avoid overfitting, while still describing possible seasonal patterns [46] and was used to predict values of *e* for each day of the sampling period.

Predicted daily values of *e* from the *gam* smoother were then used to simulate the gonad growth during the same timeframe and to explore how the changes in energy reserves and seasonal temperature affected the investment towards reproduction by *L*. *elliptica* during the years 1998 and 1999. Although DEB theory specifies that state variables (*V*, *E*, *E*_*H*_ and *E*_*R*_) cannot be measured empirically, it is possible to relate the reproduction buffer (*E*_*R*_) with the gonad tissue imposing some handling rules [47,48]. Here, some assumptions were taken to describe the gonad growth and the spawning event in summer 1999: (1) *E*_*R*_ was assumed to be always in the gonad, therefore at constant conditions of temperature, reserves level and animal size, the gonad will grow linearly as reproduction flux (*ṗ*_*R*_) is constantly transformed into gonad and accumulated [14]; (2) to account for the spawning event during 1999 a fixed date was assumed for the start of spawning (mid-December), which will last until March [24,25]; (3) only half of the gonad mass at the onset of spawning will be released during the spawning season, the rest of the gonad is resorbed and reused for further reproduction events [25,49]; (4) the released amount of gonad at a given time follows a logistic curve, which parameters were calculated based on the proportion of specimens with gonads in spawning stage [25,49] during the spawning season, allowing to take into account the variability of the spawning intensity during the spawning season; (5) the reproduction flux (*ṗ*_*R*_) is directly released (i.e. not accumulated in the gonad) during the spawning season.

To further link the observed biological traits measured in the field (gonad development, loss of weight) to available resources, we used the rates of change observed in the approximated scaled reserves (*e*) to assess food availability. In DEB, energy reserves are determined by the availability of food resources in the environment and therefore reserve changes are directly related to the products available for consumption in the environment. To account for the variability of food the DEB model uses a scaled version of the Holling’s type II functional response [50], *f*, to account for the effects of food availability on feeding (S1 File). The scaled functional response *f* directly relates to the assimilation flux *ṗ*_*A*_ and therefore provides information on the energy acquired by the organism (Eq 3, S1 File).

$${\dot{p}}_{A}=\left\{{\dot{p}}_{Am}\right\}\cdot L^{2}\cdot f$$ (3)

where *{ṗ*_*Am*_*}* is the maximum surface-area specific assimilation (Table 1)

It was possible to use *e* to approximate the scaled functional response *f*. The scaled reserves (*e*) tends to be in equilibrium with available food, when this happens *e* = *f* and therefore *e* is often used as a proxy to account for food availability [14]. This is probably not the case most of the time in *L*. *elliptica*, due to the hypothesized seasonal variability of food [24] and the slow metabolism of the organism [37,39]. At the natural temperature of their habitat, it may take months or years for polar organisms to reach equilibrium with available resources when these are kept constant [17]. It is still possible to relate *f* and *e* using the DEB theory dynamic of the reserves (S1 File). Hence, the rate of change of the reserves is directly related to food availability through Eq 4.

$$\frac{\Delta e}{\Delta t}=\left(f-e\right)\cdot \dot{\upsilon }\cdot L^{-1},$$ (4)

where $\dot{\upsilon }$ is energy conductance (Table 1).

Finite differences of the previously calculated *gam* smoother were then used to describe the change in *e* (*Δe*) by day (*Δt*). From thereon, Eq 4 was used to yield the scaled functional response *(f*). The scaled functional response cannot be quantitatively related to food density due to the lack of information on feeding and clearance rates of *L*. *elliptica* at different food densities. However, it offered a quantitative assessment of the energy assimilated by the organism during 1998–1999 and a scale of available resources that relates to animal condition and metabolism during the years 1998 and 1999 and how resources influenced the potential growth and reproductive outputs.

To gain insight in the factors that may have determined *f* during 1998 and 1999 we analysed the observed variability of *f* against potential food sources. *L*. *elliptica* is a suspension feeder, and therefore the availability of food resources depends on the nutrients available in the water column [51]. Chlorophyll concentration is often used as a proxy for quantifying food available to suspension feeders, however for a population located at 30m depth, sea surface chlorophyll concentration may not be such a good proxy in this respect [24]. In this study, we analyse the yielded *f* against surface measured chlorophyll concentration and the particulate organic carbon flux at 30m depth measured at Marian Cove [52], while still highly related to chlorophyll concentration, this flux could be a better proxy to the amount of food reaching *L*. *elliptica*. Moreover we also explored the effect of lithogenic sediment particles [52] in *f*. Lithogenic particles have no nutrient value, however they are known to compete with food particles, reducing *L*. *elliptica* feeding efficiency [21,32]. Linear models were used with the aim to test the variation of observed *f* that could be explained by chlorophyll, POC and lithogenic fluxes. Measurements of POC and lithogenic fluxes were obtained from [52] and consisted of monthly averaged rates. Chlorophyll measurements were obtained from [24] and consisted of weekly measurements on sea surface. For analysis mean *f* and chlorophyll values were calculated for the same time frame covered for each POC and lithogenic flux value given by [52]. Chlorophyll and POC were highly correlated and were never used together. Initial models considered chlorophyll or POC with lithogenic flux and their interactions. Model selection was performed by Akaike Information Criterion (AIC) and likelihood ratio test using R v.3.15 [41].

## Results

### DEB parameters

Adjusting the Arrhenius function to the respiration data from Peck et al. [39] yielded a *T*_*A*_ of 4832 K, a lower limit temperature (*T*_*L*_) of 271 K and an Arrhenius temperature at the lower limit (*T*_*AL*_) of 19660 K (see S1 File). These values were used as fixed values in the covariation method to determine the other DEB parameters. Further parametrisation was not necessary as these parameters accurately described the temperature sensitivity detected in other observations of *Laternula elliptica*.

The calculated value of the post-metamorphic shape coefficient yielded from observations of shell length and wet weight relationship was of 0.33±0.02 (mean ± sd). This value was set as free within the covariation method, which gave back a definitive value of 0.341 (Table 1).

Parameter estimations are detailed in Table 1. The total fit of the covariation method resulted in a MRE (mean absolute relative error) of 0.100. In general, the estimated model parameters accurately describe the data used for their estimation (Table 2, Fig 1). During model parametrisation, it was assumed that the observed organism condition was the result of an average food availability for the population. The scaled functional responses (*f*) in Table 1 are considered in equilibrium with the organism reserve and therefore *e* = *f*. Comparison of data from different populations allowed us to understand the food limitation for the population at Marian Cove and Potter Cove with an average scaled functional response of 0.33 and 0.38 respectively, when compared with the population at Rothera. Animals from Rothera [39] showed the best condition (length—weight and higher metabolic rate) so the food level at Rothera was taken as maximum reference, we fixed the scaled functional response for the animals in Rothera to *f* = 0.8 (instead of the possible maximum *f* = 1) because it was rather unprovable that the animals from Rothera point were fed ‘*ad libitum’* considering the seasonal variability in the area [37]. Although the effect of temperature is well described by the calculated Arrhenius curve, the DEB model consistently underestimated respiration at temperature for the organisms at Rothera (Fig 1D). *L*. *elliptica* used in these experiments were starved [39], as such we assumed that there were no contribution of assimilation to the oxygen consumption [14]. It is possible that the starvation period was not long enough and there was still some contribution from assimilation explaining the consistent underestimation.

Seasonal variability of the scaled functional response in Marian Cove was assessed in more details during the model exploration.

### Model exploration

**Fig 2:**
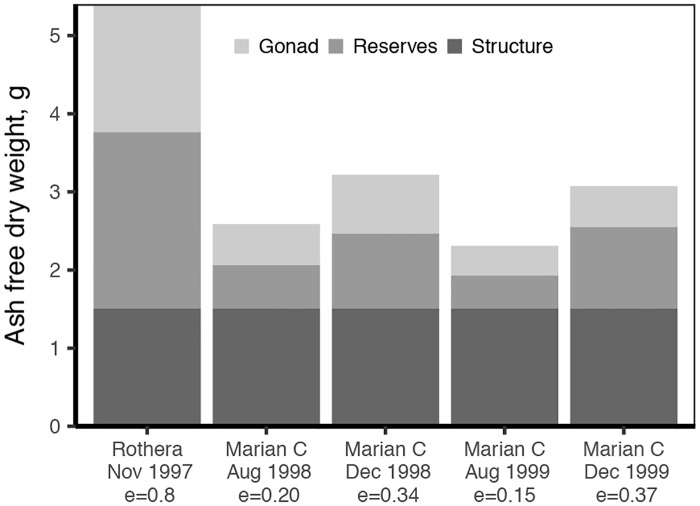
DEB state variables as biomass for different levels of scaled reserves (*e*), for a standard individual of 7.5cm shell length. Gonad weight for Rothera was probably overestimated as e = 0.8 was used for the whole gonad growth season.

DEB parameters relate to a model organism with a large storage capacity, where energy reserves of animals with no limitation in food supply compose more than 65% of the total dry mass (excluding gonads). However natural populations never fed ‘*ad libitum’* due to large variability of food resources, and their reserves are greatly reduced: that is specially the case for the animals at Marian Cove where reserves compose between 20% and 41% of total mass (excluding gonads, Fig 2). Reserves for the animals at Rothera were much higher suggesting better feeding conditions.

**Fig 3:**
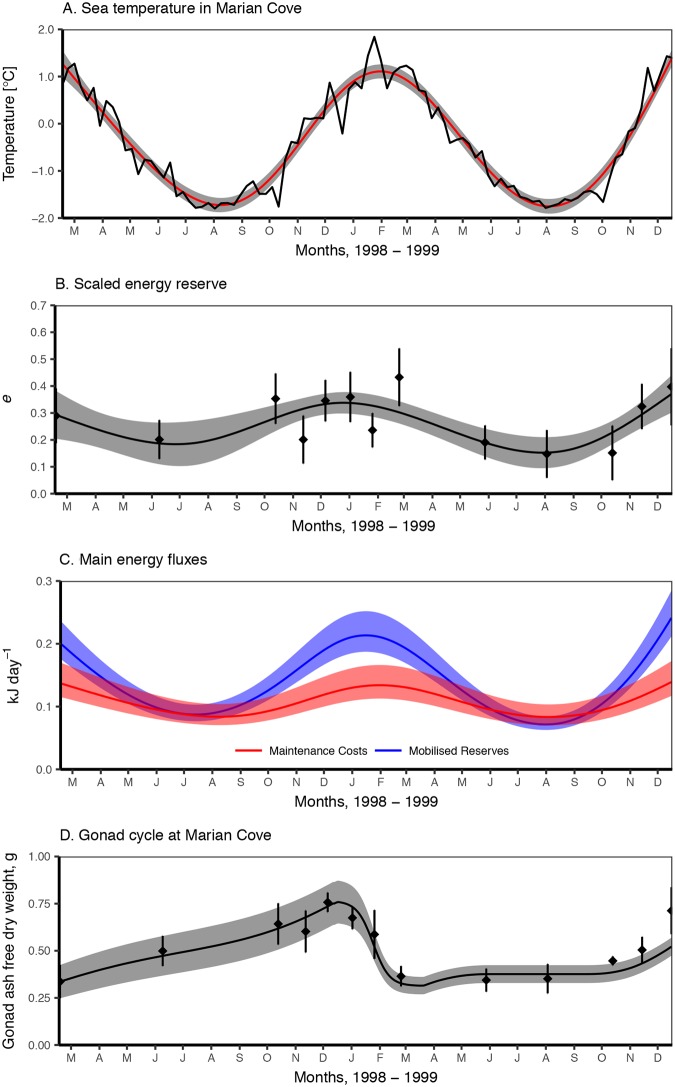
Estimated seasonal variation of energy reserves, metabolism and reconstructed gonad growth of *L*. *elliptica* at Marian Cove during 1998–1999. **A**. Temperature measured in the field. Red line is a fitted smoother (See S3 File), shaded area is the 95% confident interval (ci) of the smoother from [24]. **B**. Reconstructed scaled energy reserve. Dots and bars are mean values of *e* and the 95% ci yielded directly from field observations of length-weight (Eq 2). Line and shaded area respectively correspond to the mean and 95% ci of *gam* smoother (see Methods and S3 File). **C**. In blue calculated mobilisation flux (*ṗ*_*c*_) for current energy reserves (*e*) (see S1 File for formulation, figure 3.B). In red calculated maintenance costs, as somatic maintenance (*ṗ*_*M*_) plus maturity maintenance (*ṗ*_*J*_) for a population with shell length distribution observed by Ahn et al. [24]. Both corrected for current temperature at day (Fig 3A). **D**. Gonad ash free dry weights. Dots and bars are the mean and the 95% ci of the field observations [24]. Line and shaded areas are DEB model predictions mean and 95% ci considering a population with the same shell length distribution as used by Ahn et al. [24] and using the approximated *e* from Fig 3A. Gonad production was corrected by temperature (see S1 File).

DEB model parameters were explored further by comparing model outputs with time-series of biological traits of *L*. *elliptica* from Marian Cove, comprising the raw data collected by Ahn et al. [24] during the years 1998 and 1999 on animal condition, gonad development and seawater temperature (Fig 3). The approximation to the scaled energy (*e*) from length—weight data (using Eqs 1 and 2 and *gam* smoother S3 File) showed a seasonal variation of energy reserves during the year 1998–1999 (Fig 3B). Reserves are lower during spring and winter months, and reach their maximum during autumn-summer. Reserves varied seasonally by approximately a 50%. The minimum reserve level occurred during the winter 1999 (Figs 2 and 3B).

A description of the metabolism pattern was possible by using the approximated *e* and the estimated DEB parameters and theory to describe the main energy fluxes during 1998–1999. These fluxes relate to organism reserves, size and environmental temperature following DEB theory (S1 File). The reduction of energy reserves reduced significantly the energy mobilisation (*ṗ*_*c*_), which is directly related to the size of reserves (S1 File), during the winter months (Fig 3C). Although maintenance costs (*ṗ*_*M*_ + *ṗ*_*j*_) decreased during winter due to temperature (Fig 3C), the decrease of *ṗ*_*c*_ due to the lowered reserves was steeper and in the middle of the winter *ṗ*_*c*_ was barely enough to cover maintenance. The situation was worse during winter 1999 with reserves being lower than in 1998. In this case, the organisms were not able to cover maintenance (Fig 3C).

Further exploration of the DEB parameters and estimated *e* was done by simulating the gonad growth from March 1998 to December 1999. Predicted gonad dynamics described the observed patterns in the field (Fig 3D). Higher reserves during 1998 resulted in the gonad growing all year long (Fig 3D), with a perceptible slow down during June and July 1998, due to the decrease in energy mobilisation (Fig 3C). When *ṗ*_*c*_ is larger than maintenance the organism, can grow, and invest on reproduction, that does not happen in 1999 when there was an energy deficit, this is reflected by no increase on gonad size during the winter of 1999 (Fig 3C and 3D) as DEB prioritises maintenance over gonad growth [14]. Gonad growth accelerated during the last months of 1999 in response to the increase of *e* and temperature (Fig 3B, 3D and 3A).

**Fig 4:**
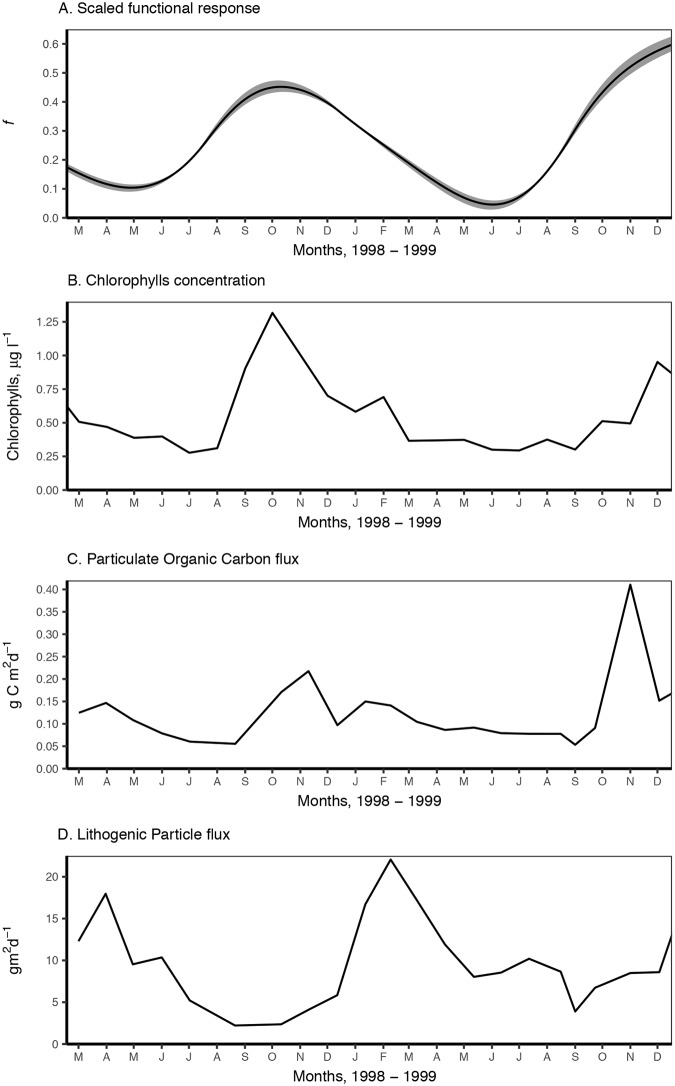
Estimated food levels for *L*. *elliptica* in Marian Cove during 1998–1999 and measured sediment fluxes (Khim et al. [52]). A. Reconstructed scaled functional response (*f*) from energy reserves dynamics considering temperature. For both, lines are mean and shaded area is the 95% ci. B. Chlorophylls concentration [24] C. Particulate organic carbon flux. D. Lithogenic particle flux. C & D. measured at Marian Cove at 30m depth. For details see Khim et al. [52].

The observed variation of reserves was analysed using DEB theory reserve dynamics (S1 File). The scaled functional response during the years 1998 and 1999 was reconstructed applying DEB reserve dynamics (Eq 4) to the finite differences in *e* given by the *gam* smoother (Fig 3B, S3 File) (Fig 4A). Reconstructed *f* (Fig 4A) showed how food resources varied seasonally between 1998–1999 with minimum values during winter and increasing fast during spring and early summer. As expected from observed *e*, *f* and therefore assimilation was lowest during winter 1999, when *f* decreased rapidly during summer and autumn and increased very fast during spring. The linear model (S3 File) considering chlorophyll concentration (Fig 4B), the amount of lithogenic particles in the sediment flux (Fig 4D) and their interaction provided the best fit for the observed *f*, explaining 45% of variability (see S3 File for model parameters and residual plots). The variability of *f* was directly related to chlorophyll concentration (S3 File, p-value < 0.01, Fig 4B) and indirectly to the lithogenic particles sedimentation (p-value < 0.01, Fig 4D). An alternative model considering POC flux (Fig 4C) instead of chlorophyll concentration and the amount of lithogenic particles in the sediment flux (Fig 4D), was the next best fit and explained 37% of the variability. The same negative effect was observed with the lithogenic flux, while POC flux was directly correlated with *f* (p-value < 0.01, S3 File).

## Discussion

The present study provides the parametrisation of a DEB model for *Laternula elliptica*, a common Antarctic suspension feeding bivalve which plays a key role both structurally and functionally in shallow Antarctic marine ecosystems [21]. DEB theory and the parameters estimated here provide a mechanistic model that can be effectively used to understand the physiological condition of *L*. *elliptica* and the inherent adaptations to its habitat. In general DEB parameters describe an organism specially adapted to the characteristics of Antarctic environment, such as year-round low temperatures and extreme seasonality in food availability [2,54]. The exploration of DEB parameters against field observations recorded for two consecutive years allowed to gain insight on the variability of resources and the seasonal metabolism of *L*. *elliptica* and promoted the characterization of the model performance against data independent of that used for parameter estimation. Moreover, this data was used to link the observed variability of resources to sedimentation fluxes.

Dynamic Energy Budget model parameters for *L*. *elliptica* assigned a large reserve (≈65% of total weight, Fig 2) enough to survive long starvation, which benefitted from the low maintenance costs (*ṗ*_*M*_) at the low-temperature conditions. Considering the temperature effect, *ṗ*_*M*_ is larger but not very different from the values for other marine bivalves (at 20°C *L*. *elliptica [ṗ*_*M*_*]* is 31.2 J·cm^-3^·d^-1^ compared with the mean for marine bivalves of ≈24.54 J·cm^-3^·d^-1^, from the “Add my pet” webportal: http://www.bio.vu.nl/thb/deb/deblab/add_my_pet/), suggesting no adaptation to reduce the maintenance costs. *L*. *elliptica* has a large capacity to mobilise energy ($\dot{\nu }$) that allows the organisms to keep a high supply of energy from the reserves even at low temperatures and when reserves are low. The energy conductance of *L*. *elliptica* at 0° is directly comparable (without correction for the low temperature) to that of organisms from habitats with a temperature close to 20°C ($\dot{\nu }\;=\;0.02\;{\text{cm d}}^{-1}$, “Add my pet” webportal). This allows *L*. *elliptica* to keep the same level of energy mobilisation at 0°C, as that of temperate organisms at 20°C, keeping activity to the comparable levels [21]. The large capacity to build reserves associated with low maintenance costs allows *L*. *elliptica* to grow fast at low temperatures and even when food is not abundant, reaching its maximum size after 9 to 10 years [26]. Another important adaptation described by a DEB parameter is the high assimilation power (*{ṗ*_*Am*_*}*) of *L*. *elliptica* (at 20°C *L*. *elliptica {ṗ*_*Am*_*}* is 399 J·cm^-2^·d^-1^ compared with the mean for marine bivalves of ≈20.5 J·cm^-2^·d^-1^, from the “Add my pet” webportal). Even if it was not possible to relate it to other characteristics such as the clearance rate or the functional response, this parameter indicates a high capacity to make use of the available food resources, allowing *L*. *elliptica* to build up reserves efficiently when food is available. It also allows for a large body as size is determined by the ratio between assimilation power and maintenance costs of structure (S1 File), with *L*. *elliptica* being one of the only two large bivalves species in Antarctica [24]. Altogether, the DEB parameters obtained in this study fit to an organism especially adapted to cold environments where food is limited being associated with extreme seasonality in day length [1].

The use of a time-series on *L*. *elliptica* condition and gonad growth alongside DEB parameters took advantage of one of the key characteristics of DEB theory and models: linking observed biological/physiological traits to environmental temperature and food resources. At the same time, we explored how the DEB parameters estimated here can assess biological traits in varying conditions. Data on weight-length and gonad development shows that energy reserves levels are seasonal (Figs 3 and 4). A longer period of food shortage happened in 1999 and animals lost a 25% of mass during the period of March through August 1999 [24]. With the use of DEB parameters on the same data the present study provided an assessment of the food available and how it relates to observed energy reserves variability. The considerable weight loss in winter 1999 related to a prolonged period of low energy uptake, during which *L*. *elliptica* uses up to 66% of its reserves (Figs 2 and 3B). Along with the severe weight loss gonad development was also affected. Gonad development seemed retarded in 1999, while during the same months in 1998 gonad was maturing continuously (Fig 3C and 3D). The high capacity to adapt to available resources by *L*. *elliptica* allowed to increase its energy reserves fast at the end of the winter resulting in a fast increase on the investment on reproduction and the consequent gonad growth at the end of 1999.

The exploration of biological traits showed that the scaled functional response (*f*) during the years 1998 and 1999 followed a seasonal pattern. The determination of *f* presented here allowed us to assess quantitatively the seasonal assimilation flux (S1 File). The assimilation flux is directly related to food quantity and quality through the scaled version of a Holling’s type II functional response [14], therefore *f* is a scaled quantification of the amount of food available and the capacity of the organism to uptake it. The results showed than *f* is never high in Marian Cove (max of 0.4 over 1) and considerably lower than that of Rothera in November 1997 (Fig 2); however, it is considerably larger in the autumn-summer periods of 1998 and 1999 (*f* = 0.35–0.4) compared to spring-winter (*f* = 0.15–0.2). This study intended to link observed *f* to field measurements of environmental variables that may be able to describe the food availability for *L*. *elliptica* and its capacity to make use of this food. *L*. *elliptica* is a suspension-feeder and animal condition and reproduction cycle have been previously linked to the spring phytoplankton bloom [24]. The results here agreed with the observations by Ahn et al. [24] as surface seasonal chlorophyll explained the seasonal food variability (Fig 4, S3 File). However, food availability is a complex variable, difficult to quantify, and chlorophyll concentration in surface water may not suppose always a good link as the amount reaching the bottom will depend on oceanographic conditions which varied for different locations, seasons, etc. Benthic suspension feeders like *L*. *elliptica* may rely primarily on benthic food materials [55]. Although these benthic food materials need to be resuspended into the water column to be accessible [26], amount of which may not be accounted by measurement of surface chlorophyll concentration. The sedimentation flux of particulate organic carbon (POC) was also a good proxy to *L*. *elliptica* scaled functional response, POC flux in Marian Cove being related to the phytoplankton bloom [52] however, it was measured at the depth where the animals were sampled and it also considers sedimentation of resuspended particles. However, chlorophyll and POC flux were not the only factors describing *L*. *elliptica* seasonal feeding, the analysis of the sediment fluxes found a strong negative effect of the lithogenic particle flux on *L*. *elliptica* scaled functional response. A negative effect of sediment load on condition and growth of *L*. *elliptica* have been reported before in the field [32] and a reduction in assimilation has been assessed in experiments [22]. *L*. *elliptica* feeds while filtering the water, so, when the concentration of particles without nutrition value increase relatively to the particulate carbon the effective concentration of food decreases, in the extreme cases a high concentration of particles may clog the filtering system [22,56,57]. Although two years may not be a long enough time-series to establish robust correlations, the results clearly highlight the importance of nourishing mass as well as its concentration within the total suspended particles, showing that the effects of sediment run-off are already affecting the seasonal feeding of *L*. *elliptica* population in Marian Cove. Establishing a link of nutritional state and assimilation of *L*. *elliptica* with variables such as POC is important to understand how environmental variations affect *L*. *elliptica* performances and to describe differences among populations. The difference between Marian Cove and Rothera *e* was due to different levels of food that could be related to a larger different primary production, and therefore of POC flux between the locations and also for a smaller lithogenic flux in Rothera as it is not influenced by a land-terminating glacier [2,9,24,58].

*Laternula elliptica* is a key organism in Antarctic shallow soft bottoms. Its capacity to enhance the carbon flux to the sediment by the biodeposition of faeces and pseudofaeces [21] creates an enriched area and reduces suspended matter that sustains associated benthic biota [22]. As such, the performance and population dynamics of *L*. *elliptica* has consequences for its ecosystem. Exploring the DEB model allowed to show how the life of *L*. *elliptica* was affected by the balance between the efficient use of food available during spring-summer season and the starvation during a food depleted winter, due to the bivalve’s low maintenance metabolism. Antarctic coastal ecosystems are changing fast, especially in the Western Antarctic Peninsula [6]. Because of global warming, ice dynamics are already changing. The land glacier retreat and the decrease of sea-ice season duration [7] induce a cascade effect on coastal environments, decreasing salinity, increasing ice scouring and land sediment run-off [58,59]. All these changes are affecting primary production, POC fluxes, disturbance, resuspension and sediment load in coastal waters [9,58–60] and therefore they are changing the dynamics of the food abundance for *L*. *elliptica*. If food in summer is available in a quantity or long enough to build up a reserve large enough it has the potential to jeopardize the capacity of *L*. *elliptica* to survive winter starvation. The decrease observed in 1999 already resulted in a significant mass loss and slowed down gonad production (Fig 3B and 3D), if that situation was to be prolonged, reproduction in 1999 may have been affected too. Moreover, Antarctic coastal bottom water is also warming up as a consequence of global change, although the rate of change is small compared with that of air temperature [5,6], a raise in temperature during winter with very low food will increase the maintenance costs while decreasing the time *L*. *elliptica* can withstand starvation (Fig 3C). On the contrary, an increase of food availability will improve condition, favouring growth and reproduction and buffering any non-lethal effect of warming.

### Conclusion

Key species have a large impact on their ecosystems which rely on them for provision of food, space or protection among others [47,61,62]. In this context, knowledge on the physiological performance of key species under varying environmental conditions is fundamental to understand how such variations might affect the ecosystem. Changes in physiological performance will result in changes on key species population dynamics, affecting recruitment and/or survival [63,64] and particularly species key functions that will impact ecosystem structure and functioning [65]. In the case of *Laternula elliptica*, changes of its population density or feeding activity will impact bentho-pelagic coupling function and therefore will have a cascade effect on the biota that depends on the sediment enrichment. The DEB parameters estimated in this study provide detailed information on the metabolic strategy of *L*. *elliptica* and provided a mechanistic link between the organism’s physiology and its environment. DEB parameters successfully describe the observed field variation of the population in condition and allocation to gonad, becoming a powerful and robust tool to understand the effects of varying environments on *L*. *elliptica* performance. Further knowledge on filtration feeding and deposition rates at different densities of food will allow to use this DEB model to assess the impact of *L*. *elliptica* through quantification of its function as a bentho-pelagic coupler. Moreover, this study provides a link between organism condition and energy uptake and important environmental variables: chlorophyll, POC and lithogenic fluxes which are important oceanographic variables for which large scale measurements and estimations are available. This link allows to derive *L*. *elliptica* performance directly from knowledge on these fluxes and temperature making it possible to express organism biological traits in a spatially-explicit context, to develop mechanistic species distribution models [66], which can be applied to study a range of present and future scenarios to predict future species performance and distribution.

## Supporting information

S1 File General description of standard DEB model assumptions and notation. (PDF)

S2 File Map with the localities mentioned. (PDF)

S3 File Statistical models summaries and residual plots. (PDF)
