# Supplementary material for: A Dynamic Energy Budget (DEB) model to describe Laternula elliptica (King, 1832) seasonal feeding and metabolism
Source: PLoS One. 2017 Aug 29;12(8):e0183848. doi: 10.1371/journal.pone.0183848 (PMC5574559; doi:10.1371/journal.pone.0183848)
Supplement: S1 File — (PDF) [file pone.0183848.s001.pdf]

A Dynamic Energy Budget (DEB) to describe *Laternula elliptica* (King, 1832) seasonal feeding and metabolism

Antonio Agüera*[1], In-Young Ahn[2], Charlène Guillaumot[1] and Bruno Danis[1]
[1] Laboratoire de Biologie Marine CP160/15. Université Libre de Bruxelles, F. D. Roosevelt 50, 1050 Brussels, Belgium
[2] Korea Polar Research Institute (KOPRI), 26 Sondomirae-ro, Yeonsu-gu, Incheon 21990, Republic of Korea
* corresponding author e-mail: antonio.aguera@gmail.com

S1: Dynamic Energy Budget theory and models:

This supplement aims to describe the background information underlying the standard DEB model serving to understand the discussion and methods of the main manuscript. Further details on DEB theory and standard and non-standard DEB models can be found in references (Kooijman, 2010; van der Meer, 2006; van der Veer et al., 2006).

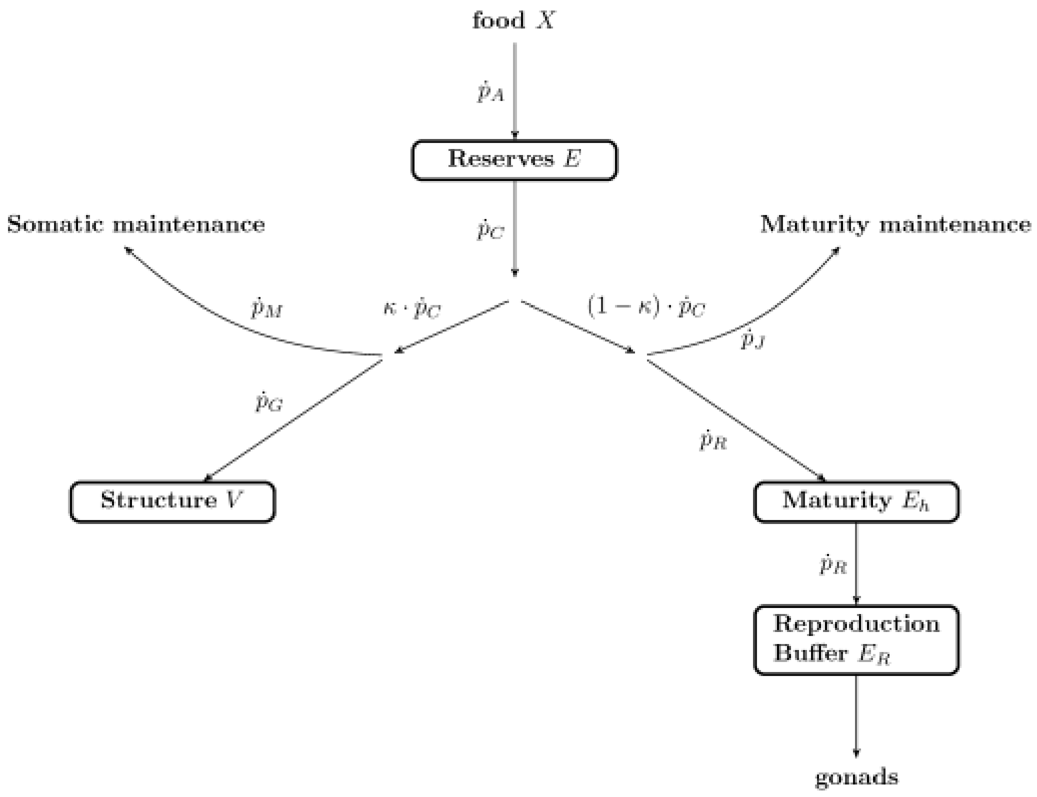

Figure 1. Schematic representation of standard DEB model. Arrows represent energy fluxes (J d^{-1}) that drive the dynamics of the four state variables (boxes). Energy enters the organism as food (X), is assimilated at a rate of $\dot{p}_A$ into reserves (E) . Mobilization rate ($\dot{p}_C$), regulates the energy leaving the reserve to cover somatic maintenance ($\dot{p}_M$), structural growth ($\dot{p}_G$), maturity maintenance ($\dot{p}_J$), maturation ($\dot{p}_R$) (immature individuals) and reproduction ($\dot{p}_R$) (mature individuals). κ is the proportion of the mobilised energy diverted to $\dot{p}_M$ and $\dot{p}_G$, while the rest is used for $\dot{p}_J$ and $\dot{p}_R$.

The DEB model assumes that an individual's physiological processes are dependent on surface area or body volume. The model conceptually discriminates between three state variables: Energy reserves (E), structural volume (V) and maturation (E_H); once the individual reaches puberty another state

variable is included: reproductive buffer (E_R). The mass of an organism at any point is given by the contribution of reserves, structural volume and reproductive buffer. Maturation however is understood as energy that is dissipated or spent by the organism in the increase of maturity, as such this state variable does not contribute to total mass, and once the maximum maturity (puberty) level is achieve the energy allocated to maturation starts to be used in reproduction. DEB theory assumes that these state variables are not directly measurable (empirically), however the DEB model fully describes their dynamics by a set of equations that characterize the individual's physiological condition (van der Meer, 2006).

In the DEB context, all assimilated products first enter the reserve pool. From the reserves pool, energy is allocated to growth, reproduction and maintenance. The κ-rule says that a fixed fraction (κ) is allocated to maintenance and growth while the rest (1-κ) is available for development (maturity) and reproduction. The DEB model explicitly incorporates the existence of overhead costs associated to the processes of energy conversion as this is mobilized among compartments. Those overhead costs are linked to assimilation, growth and reproduction and translate as energy losses (figure 1).

One of the main DEB assumptions is that organisms are isomorphs, i.e. their shape does not change as they grow. The model relies on structural length L (cm), rather than physical length L_W (cm), to provide a measure of size. Structural length relates to structural volume (V) discriminating between contributions from other state variables. Also structural length is not affected by the organism's shape, thus favoring inter-species comparisons (Kooijman, 2010). The DEB parameter shape coefficient δ_M (dimensionless) serves to translate physical measurements taken from some representative length (e.g. arm length) to structural length $L = \delta_M \cdot L_W$. In the DEB model, several energetic processes are dependent either on surface area (L^2, cm^2) or on structural volume (L^3, cm^3) and, as such, several parameters are either scaled by volume or area.

DEB theory makes use of a specific nomenclature: all rates (units t^{-1}) are written with a single dot diacritic, e.g. $\dot{p}_A$; all surface-area specific quantities (units cm^{-2}) are written in curly brackets, e.g. $\{\dot{p}_{Am}\}$; and all volume-specific quantities (units cm^{-3}) are written in square brackets, e.g. $[\dot{p}_M]$.

Energy Reserve dynamics:

Energy reserves are determined by the availability of food resources in the environment, in that way reserves are in equilibrium with the products available for consumption in the environment. Consequently their dynamics are directly related to the environment. The DEB model uses a scaled version of the Holling's type II functional response (Holling, 1959), f, to account for the effects of food availability on feeding and assimilation flux ($\dot{p}_A$):

$$\dot{p}_A = \{\dot{p}_{Am}\} \cdot L^2 \cdot f \text{ , where } f = \frac{X}{X+X_K} \qquad (1)$$

$\{\dot{p}_{Am}\}$ is the maximum area-specific assimilation rate (J d^{-1}cm^{-2}). X is the amount of available resources as density (by area or volume depending on the organism), X_k represents the half-saturation coefficient (density at which feeding rate is half of its maximum value). f is not invariable, the functional response will change when the resource (X) is different. f may also change between life stages. For example, the pelagic larva feeds on a resource for which density is estimated by volume unit, while the adult preys or grazes on a resource which density is estimated by area unit.

Energy is mobilized ($\dot{p}_C$) from the reserves to cover all the metabolic needs of the organism. In that manner the dynamics of the reserves are given by:

$$\frac{dE}{dt} = \dot{p}_A - \dot{p}_C \qquad (2)$$

where, $\dot{p}_C$ is the mobilization rate, given by:

$$\dot{p}_C = E \cdot \frac{\dot{\upsilon}\cdot[E_G]\cdot L^2 + \dot{p}_M}{\kappa\cdot E + [E_G]\cdot L^3} \quad (3)$$

Here two more parameters are introduced, the energy conductance $\dot{\upsilon}$ (cm d^{-1}) and the volume-specific cost of structure $[E_G]$ (J cm^{-3}). Equation (3) was derived assuming that the mobilization of reserve density is proportional to the amount of reserve density [3], which gives DEB the capacity to filter the effects of the environment on the organism's dynamics. Meanwhile assimilation rate relates to the resources available in the environment. After mobilization $\dot{p}_C$ is distributed following the κ-rule as explained before.

Somatic maintenance and growth

In ectotherms somatic maintenance costs are only proportional to structural volume [1] and can be described by the equation as follows:

$$\dot{p}_M = [\dot{p}_M] \cdot L^3 \quad (4)$$

where $[\dot{p}_M]$ is known as volume-specific somatic maintenance costs (J d^{-1} cm^{-3}). DEB prioritises maintenance; therefore, the energy available for growth will be the energy remaining after the maintenance costs have been paid:

$$\dot{p}_G = \kappa \cdot \dot{p}_C - \dot{p}_M \quad (5)$$

Growth is given as a change in the structure (excluding changes in dimensions associate to the changes in reserves or the accumulation of the reproduction buffer) (Jusup et al., 2011). DEB uses a growth formulation equivalent to the traditional von Bertalanffy, a growth model with a mechanistic basis. The von Bertalanffy growth rate, $\dot{r}_B$ (d^{-1}) describes the decrease in growth rate as the individual approximates its ultimate size (L_∞) that results from the balance between the food assimilation (surface-specific) and the somatic maintenance (volume-specific) (van der Meer, 2006). In DEB $\dot{r}_B$ is given by:

$$\dot{r}_B = \frac{1}{3} \cdot \left[\frac{\dot{p}_M}{\kappa\cdot f\cdot[E_M]\cdot[E_G]}\right] \quad (6)$$

Growth at a given size is concurrently:

$$\frac{dL}{dt} = \dot{r}_B \cdot (L_\infty - L) \quad (7)$$

Maturation and reproduction buffer

The remaining mobilized energy (1-κ) is allocated to the increase of maturity, and after the individual reached puberty to the maintenance of such maturity and reproduction. Maturity maintenance ($\dot{p}_J$, J d^{-1}) accounts for the maintenance of increased complexity attained through development and is proportional to the level of maturity. In DEB, it is given by:

$$\dot{p}_J = \dot{k}_J \cdot E_H \quad (8)$$

where $\dot{k}_J$ is the maturity maintenance rate coefficient (d^{-1}). When puberty is reached ($E_H \geq E_H^P$), maturity stops increasing and its maintenance is constant. Again maintenance is given preference, and the remaining energy is then allocated to the reproduction buffer:

$$\dot{p}_R = (1 - \kappa) \cdot \dot{p}_C - \dot{p}_J \quad (9)$$

While $\dot{p}_R$ is the rate of change in the maturation state variable (E_H) before puberty, it also describes the dynamics of the allocation to the reproduction buffer state variable (E_R) after puberty. Gonadal tissue is then synthetized following species specific rules from this reproductive buffer.

Maturation is a somewhat abstract state variable and the DEB model refers to it to determine the maturity level at any time. Maturity levels or thresholds can be set at different developmental stages (hatching, birth, metamorphosis (one or several), puberty) and the DEB model can use those thresholds as a metric for developmental stages (Lika et al., 2011).

Temperature correction for temperature-dependent rates

Physiological rates are temperature-dependent and as such they change with the environment temperature when considering ectotherms. Therefore, these rates need to be corrected accordingly. The DEB model uses the Arrhenius concept of enzyme activation to incorporate the effect of temperature in the physiological rates [1]. Arrhenius temperature (T_A) provides information on the variation of rates with temperature. The DEB model uses a curve for temperature sensitivity given by 3 parameters,

$$\dot{k}(T) = \dot{k}_1 \cdot exp\left\{\frac{T_A}{T_1} - \frac{T_A}{T}\right\} \cdot \left(1 + exp\left\{\frac{T_{AL}}{T} - \frac{T_{AL}}{T_L}\right\}\right)^{-1} \qquad (10)$$

where $\dot{k}(T)$ is the value of the physiological rate at a given temperature T (K) and $\dot{k}_1$ is the known value of such rate at a reference temperature (T_1). T_A represents sensitivity within the tolerance limits, T_L give the lower temperature tolerance limit and T_{AL} provide the sensitivity to temperature when values are close to the lower limit (Freitas et al., 2007).

Freitas, V., Campos, J., Fonds, M., Van der Veer, H.W., 2007. Potential impact of temperature change on epibenthic predator-bivalve prey interactions in temperate estuaries. J. Therm. Biol. 32, 328–340. doi:10.1016/j.jtherbio.2007.04.004

Holling, C.S., 1959. Some Characteristics of Simple Types of Predation and Parasitism. Can. Entomol. 91, 385–398. doi:10.4039/Ent91385-7

Jusup, M., Klanjscek, T., Matsuda, H., Kooijman, S. a L.M., 2011. A full lifecycle bioenergetic model for bluefin tuna. PLoS One 6. doi:10.1371/journal.pone.0021903

Kooijman, S.A.L.M., 2010. Dynamic Energy Budget theory for metabolic organisation, Third. ed. Cambridge University Press, Cambridge.

Lika, K., Kearney, M.R., Freitas, V., van der Veer, H.W., van der Meer, J., Wijsman, J.W.M., Pecquerie, L., Kooijman, S. a L.M., 2011. The "covariation method" for estimating the parameters of the standard Dynamic Energy Budget model I: Philosophy and approach. J. Sea Res. 66, 270–277. doi:10.1016/j.seares.2011.07.010

van der Meer, J., 2006. An introduction to Dynamic Energy Budget (DEB) models with special emphasis on parameter estimation. J. Sea Res. 56, 85–102. doi:10.1016/j.seares.2006.03.001

van der Veer, H.W., Cardoso, J.F.M.F., van der Meer, J., 2006. The estimation of DEB parameters for various Northeast Atlantic bivalve species. J. Sea Res. 56, 107–124. doi:10.1016/j.seares.2006.03.005
