# Supplementary material for: A Dynamic Energy Budget (DEB) model to describe Laternula elliptica (King, 1832) seasonal feeding and metabolism
Source: PLoS One. 2017 Aug 29;12(8):e0183848. doi: 10.1371/journal.pone.0183848 (PMC5574559; doi:10.1371/journal.pone.0183848)
Supplement: S2 File — (PDF) [file pone.0183848.s002.pdf]

A Dynamic Energy Budget (DEB) to describe *Laternula elliptica* (King, 1832) seasonal feeding and metabolism

Antonio Agüera*[1], In-Young Ahn[2], Charlène Guillaumot[1] and Bruno Danis[1]
[1] Laboratoire de Biologie Marine CP160/15. Université Libre de Bruxelles, F. D. Roosevelt 50, 1050 Brussels, Belgium
[2] Korea Polar Research Institute (KOPRI), 26 Sondomirae-ro, Yeonsu-gu, Incheon 21990, Republic of Korea
* corresponding author e-mail: antonio.aguera@gmail.com

S3: Location of sampling stations: Marian Cove, Potter Cove and Rothera

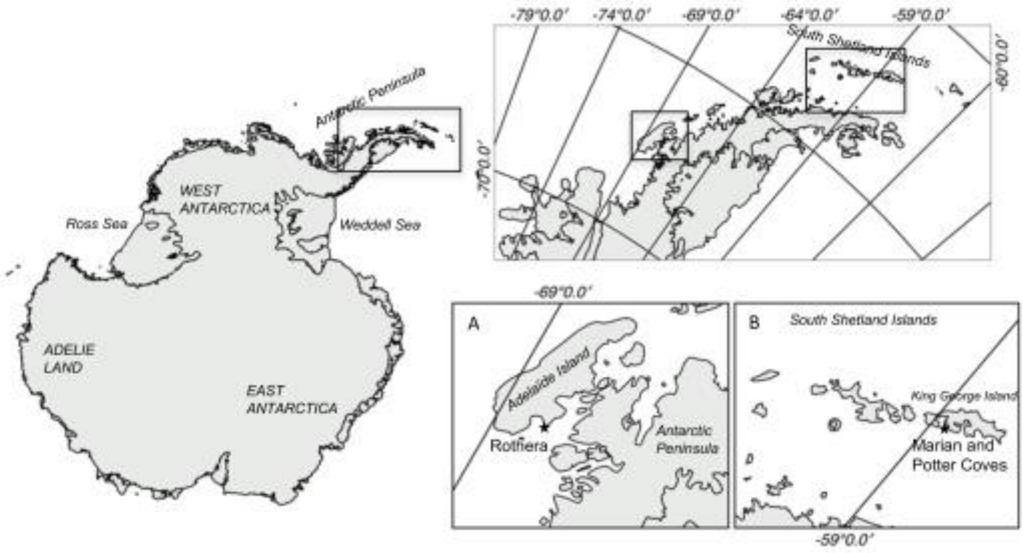

This study focus on samples taken by Ahn et al [1,2] in Marian Cove. Growth data comes from a several populations from Potter Cove [3]. Marian and Potter coves are in King George Island, they are coves within Maxwell Bay. Both these locations are characterised by being influenced by the retreat of land based glaciers, and are therefore subject to several stressors [4], including considerable sediment load from the glacier and land run-off [5,6]. Oxygen consumption at temperature data was obtained from experimental observation made by Peck et al. [7] in animals captured at Rothera point, these animals exhibited a much better condition than the ones from King George Island. Rothera is not affected by land glacier retreat and is a highly productive region [8], however data from this location was limited to average length-weight and oxygen consumption, making difficult any further comparison.

1. Ahn I-Y, Surh J, Park Y-G, Kwon H, Choi K-S, Kang S-H, et al. Growth and seasonal energetics of the Antarctic bivalve *Laternula elliptica* from King George Island, Antarctica. Mar Ecol Prog Ser. 2003;257: 99–110. doi:10.3354/meps257099
2. Ahn I-Y, Shim JH. Summer metabolism of the Antarctic clam, *Laternula elliptica* (King and Broderip) in Maxwell Bay, King George Island and its implications. J Exp Mar Bio Ecol. 1998;224: 253–264. doi:10.1016/S0022-0981(97)00201-3
3. Urban H-J, Mercuri G. Population dynamics of the bivalve *Laternula elliptica* from Potter Cove, King George Island, South Shetland Islands. Antarct Sci. 1998;10: 153–160. doi:10.1017/S0954102098000200

4. Massom RA, Stammerjohn SE. Antarctic sea ice change and variability e Physical and ecological implications. Polar Sci. 2010;4: 149–186. doi:10.1016/j.polar.2010.05.001
5. Philipp EER, Husmann G, Abele D. The impact of sediment deposition and iceberg scour on the Antarctic soft shell clam *Laternula elliptica* at King George Island, Antarctica. Antarct Sci. 2011;23: 127–138. doi:10.1017/S0954102010000970
6. Moon H-W, Mohd W, Hussin RW, Kim H-C, Ahn I-Y. The impacts of climate change on Antarctic nearshore mega-epifaunal benthic assemblages in a glacial fjord on King George Island: Responses and implications. Ecol Indic. 2015;57: 280–292. doi:10.1016/j.ecolind.2015.04.031
7. Peck LS, Pörtner HO, Hardewig I. Metabolic Demand, Oxygen Supply, and Critical Temperatures in the Antarctic Bivalve *Laternula elliptica*. Physiol Biochem Zool. 2002;75: 123–133. doi:10.1086/340990
8. Clarke A, Meredith MP, Wallace MI, Brandon MA, Thomas DN. Seasonal and interannual variability in temperature, chlorophyll and macronutrients in northern Marguerite Bay, Antarctica. Deep Res Part II Top Stud Oceanogr. 2008;55: 1988–2006. doi:10.1016/j.dsr2.2008.04.035
