# Supplementary material for: A Dynamic Energy Budget (DEB) model to describe Laternula elliptica (King, 1832) seasonal feeding and metabolism
Source: PLoS One. 2017 Aug 29;12(8):e0183848. doi: 10.1371/journal.pone.0183848 (PMC5574559; doi:10.1371/journal.pone.0183848)
Supplement: S3 File — (PDF) [file pone.0183848.s003.pdf]

A Dynamic Energy Budget (DEB) to describe *Laternula elliptica* (King, 1832) seasonal feeding and metabolism

Antonio Agüera*[1], In-Young Ahn[2], Charlène Guillaumot[1] and Bruno Danis[1]
[1] Laboratoire de Biologie Marine CP160/15. Université Libre de Bruxelles, F. D. Roosevelt 50, 1050 Brussels, Belgium
[2] Korea Polar Research Institute (KOPRI), 26 Sondomirae-ro, Yeonsu-gu, Incheon 21990, Republic of Korea
* corresponding author e-mail: antonio.aguera@gmail.com

S3: Statistic Models summaries and validation plots

GAM smoother on animal scaled energy reserves:

Family: gaussian
Link function: identity

Formula:
e ~ s(days, bs = "cr", k = 5)

Parametric coefficients:

	Estimate	Std. Error	t value	Pr(>|t|)
(Intercept)	0.27811	0.01237	22.49	<2e-16 ***

Signif. codes: 0 '***' 0.001 '**' 0.01 '*' 0.05 '.' 0.1 ' ' 1

Approximate significance of smooth terms:

	edf	Ref.df	F	p-value
s(days)	3.935	3.997	7.841	6.55e-06 ***

Signif. codes: 0 '***' 0.001 '**' 0.01 '*' 0.05 '.' 0.1 ' ' 1

R-sq.(adj) = 0.167   Deviance explained = 18.9%
GCV = 0.023717 Scale est. = 0.022937 n = 150 AIC = -133.72

Residual graphs:

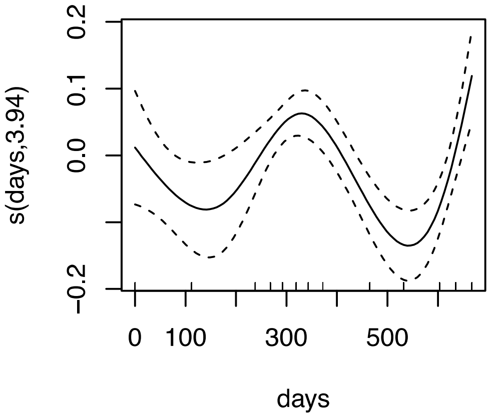

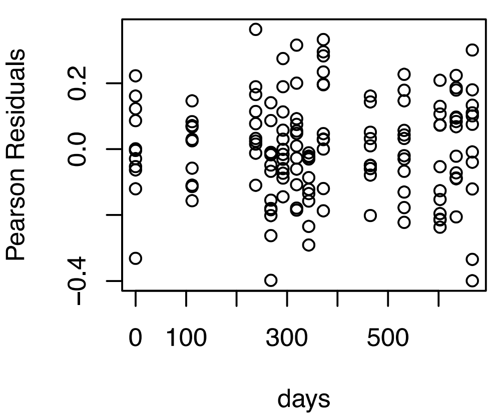

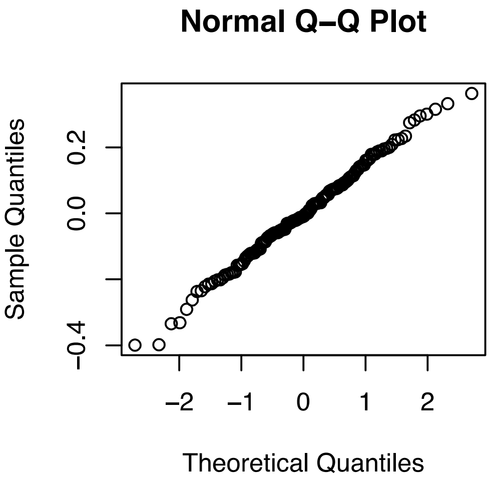

Linear model for chlorophyll concentration and lithogenic flux

lm(formula = *f* ~ chlorophyll concentration * lithogenic flux, data = MC.Env)

Residuals:

Min	1Q	Median	3Q	Max
-0.16535	-0.06584	-0.01805	0.05584	0.32501

Coefficients:

	Estimate	Std. Error	t value	Pr(>|t|)
(Intercept)	0.384863	0.150205	2.562	0.0202 *
chl	-0.003163	0.223395	-0.014	0.9889
litho	-0.045506	0.018855	-2.413	0.0274 *
chla:litho	0.068044	0.031585	2.154	0.0459 *

Signif. codes: 0 '***' 0.001 '**' 0.01 '*' 0.05 '.' 0.1 ' ' 1

Residual standard error: 0.1362 on 17 degrees of freedom
Multiple R-squared: 0.538,	Adjusted R-squared: 0.4564
F-statistic: 6.598 on 3 and 17 DF, p-value: 0.00371

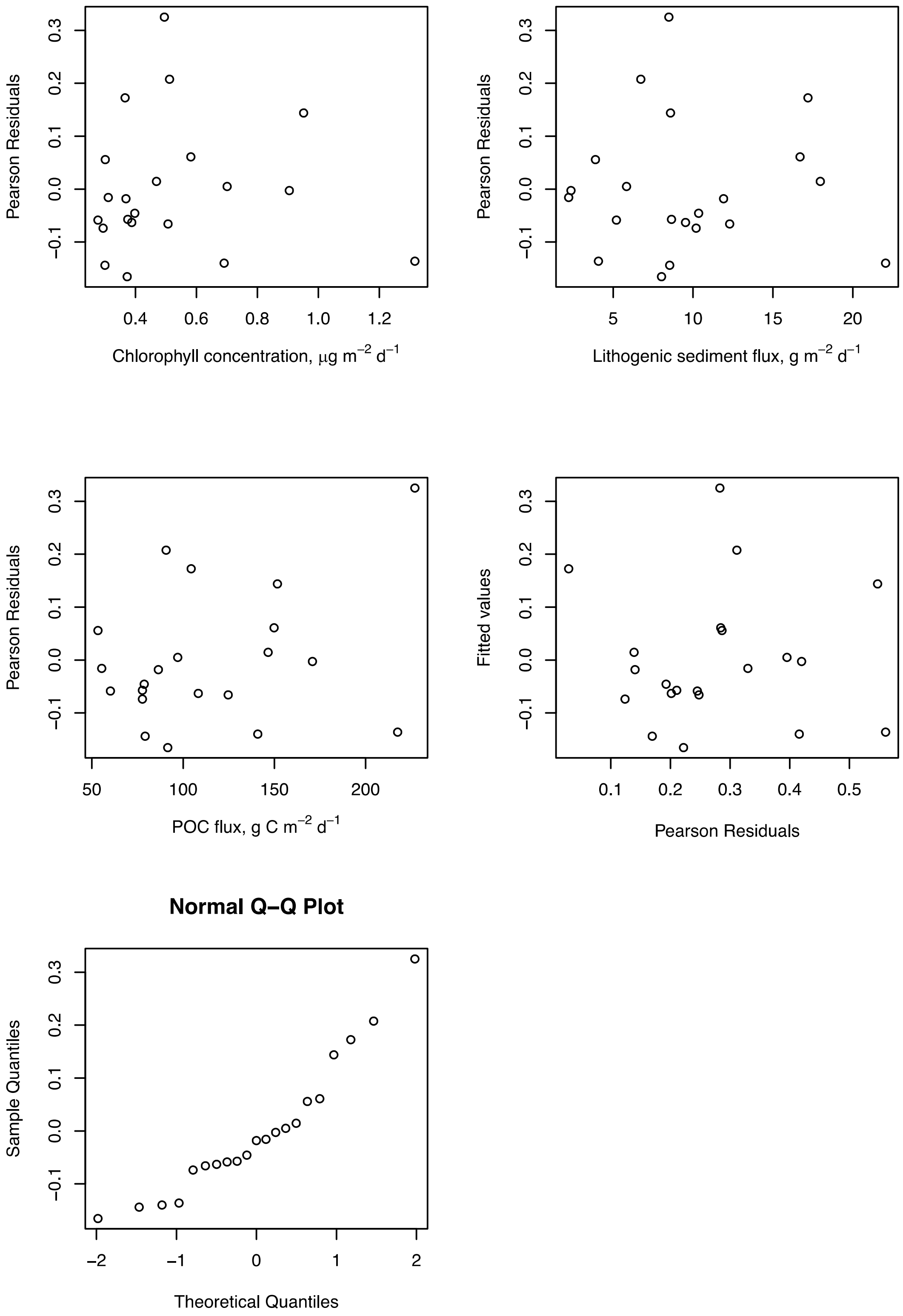

Pearson Residuals
Chlorophyll concentration, μg m^{-2} d^{-1}
Pearson Residuals
Lithogenic sediment flux, g m^{-2} d^{-1}
Pearson Residuals
POC flux, g C m^{-2} d^{-1}
Fitted values
Pearson Residuals
Normal Q–Q Plot
Sample Quantiles
Theoretical Quantiles

Linear model for particulate organic carbon (POC) and lithogenic fluxes

Call:
lm(formula = f ~ POC + lithogenic flux, data = MC.env)

Residuals:

Min	1Q	Median	3Q	Max
-0.18587	-0.08977	-0.03641	0.08134	0.31807

Coefficients:

	Estimate	Std. Error	t value	Pr(>|t|)	
(Intercept)	0.1336598	0.0946906	1.412	0.17514	
POC	0.0022943	0.0006703	3.423	0.00303	**
litho	-0.0126068	0.0062184	-2.027	0.05770	.

Signif. codes: 0 '***' 0.001 '**' 0.01 '*' 0.05 '.' 0.1 ' ' 1

Residual standard error: 0.1461 on 18 degrees of freedom
Multiple R-squared: 0.4377, Adjusted R-squared: 0.3753
F-statistic: 7.007 on 2 and 18 DF, p-value: 0.005616

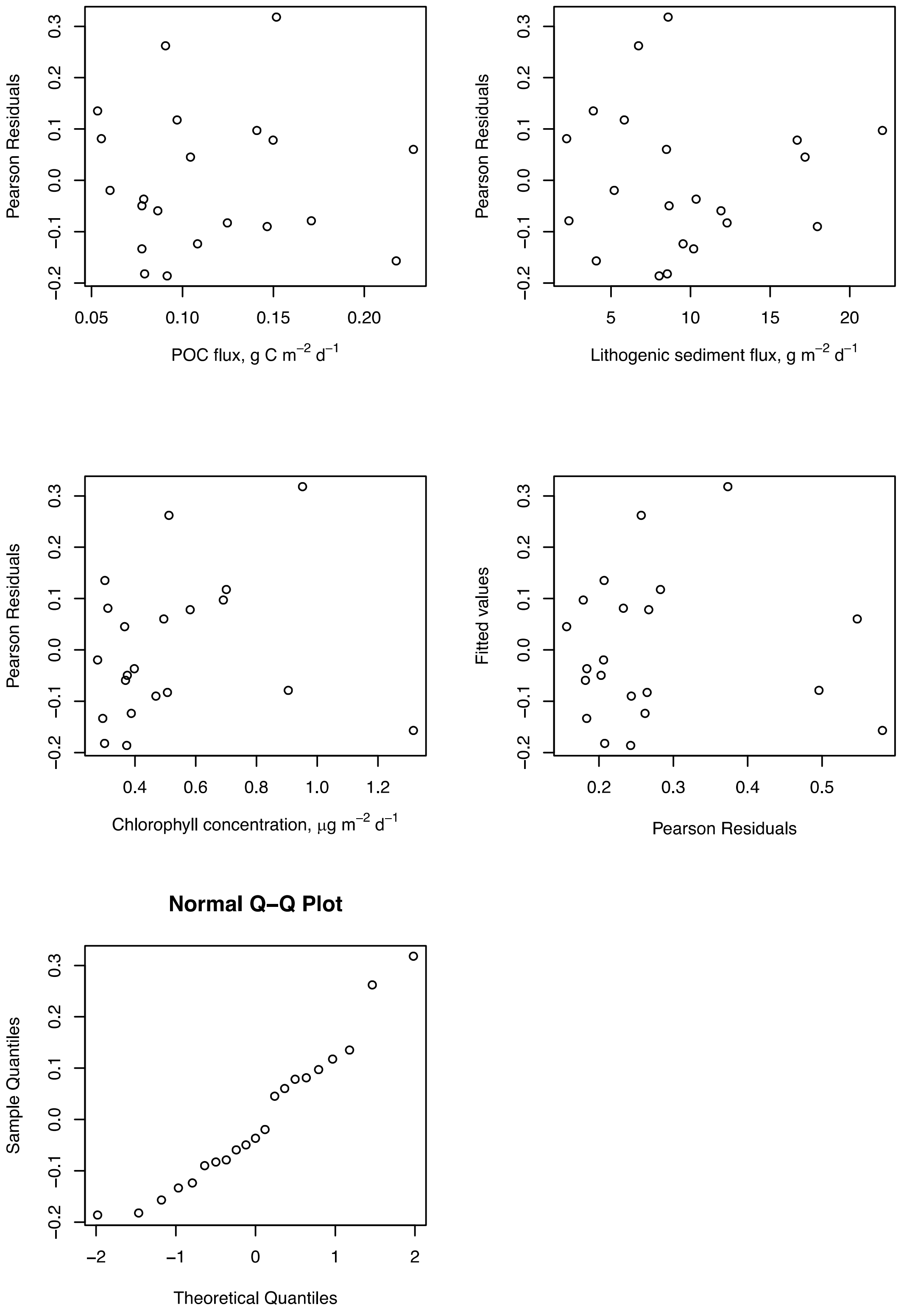
Pearson Residuals
POC flux, g C m^{-2} d^{-1}
0.05 0.10 0.15 0.20
−0.2 −0.1 0.0 0.1 0.2 0.3
Pearson Residuals
Lithogenic sediment flux, g m^{-2} d^{-1}
5 10 15 20
Pearson Residuals
Chlorophyll concentration, μg m^{-2} d^{-1}
0.4 0.6 0.8 1.0 1.2
Fitted values
Pearson Residuals
0.2 0.3 0.4 0.5
Normal Q–Q Plot
Sample Quantiles
Theoretical Quantiles
−2 −1 0 1 2
